# Arrhythmogenic right ventricular cardiomyopathy secondary to adipose infiltration as a cause of episodic collapse in a horse

**DOI:** 10.1186/s13620-015-0052-3

**Published:** 2015-10-19

**Authors:** Alexandra G. Raftery, Nuria C. Garcia, Hal Thompson, David GM Sutton

**Affiliations:** Weipers Centre Equine Hospital, University of Glasgow, Bearsden Road, Glasgow, G611QH UK; Department of Pathology, University of Glasgow, Bearsden Road, Glasgow, G611QH UK

**Keywords:** Collapse, Cardiomyopathy, Polycythaemia, Adipose infiltration

## Abstract

A 15-year-old Clydesdale cross gelding was investigated and managed over a 2-year period for intermittent collapse. The horse presented initially after an observed episode of collapse at rest, and had a resting tachycardia, elevated Cardiac Troponin I and polycythaemia. Multiple dysrhythmias were detected on telemetric electrocardiography. Vital parameters, cardiac rhythm and red cell count returned to reference range with prolonged rest but further resting syncopal episodes were observed, and due to safety concerns and limited treatment options the horse was euthanased. Post mortem evaluation identified extensive infiltration and replacement of right and left ventricular myocardial fibres with adipose and fibrous tissue, consistent with arrhythmogenic right ventricular cardiomyopathy. This report provides further information regarding the clinical and pathological features of this rarely reported condition.

## Background

Arrhythmogenic right ventricular cardiomyopathy (ARVC) is a primary heart muscle disease well documented in humans and boxer dogs for which 30–50 % of cases may have a familial component [1, 25]. ARVC is characterised morphologically by fibrofatty infiltration of the right ventricle myocardium which leads to conduction abnormalities [25]. In boxer dogs presenting signs include sudden death, ventricular arrhythmias of suspected right ventricular origin, syncope and heart failure [1]. ARVC due to fibrofatty infiltration has been previously reported in two horses as a cause of cardiac death [13] although a genetic predisposition has not been identified in equids.

Syndromes of collapse are rare in the horse but cardiac disease has been identified as the most common cause with dysrhythmias (3^rd^ degree atrioventricular block, atrial tachycardia with advanced second degree AVB and atrial fibrillation) and cardiac disease (right sided heart failure) reported [16]. Other causes include neurocardiogenic syncope, sleep disorders, seizures, hypoglycaemia, hyperkalaemic periodic paralysis and dysautonomia [15]. The presenting sign in this case was syncopal episodic collapse, with presumed transient losses of consciousness and postural tone due to cerebral hypoperfusion, followed by spontaneous recovery. In this report the presumptive relationship between syncopal episodes, clinically detected cardiac abnormalities and pathological evidence of ARVC in a 15 years old Clydesdale cross gelding is discussed, with further evidence to suggest ARVC as a rare differential diagnosis for episodic collapse in horses.

## Case presentation

A 15-year-old Clydesdale cross gelding presented to the Weipers Centre Equine Hospital for further investigation of three observed episodes of collapse in the preceding 9 months with two having occurred in the 6 weeks prior to referral. In the first observed event the horse tilted its head, developed a transient reduction in awareness and dropped saliva from its mouth. The two subsequent episodes were more marked with loss of awareness and partial loss of postural tone requiring the horse to lean against a wall to prevent full collapse (see additional resource for video). All events had occurred at rest, with rapid recovery and with no post-ictal period. Clinical examination after each event had been unremarkable. On the day of the most recent collapsing episode a blood sample was taken and a haematology profile identified a significant polycythaemia (51.1 (reference interval, 31–35) % [19]) with a mild leukopaenia (4.12 (reference interval, 5.4–14.3) ×10^9^ cells/l) and left shift neutropaenia (2.2 (reference interval, 2.7–6.8) × 10^9^ cells/l). The polycythaemia did not reflect a state of hypovolaemia as total serum protein (71 (reference interval, 60–80) g/l) and renal parameters were not significantly elevated (urea 7.2 (reference interval, < 6.84) mmol/l); creatinine 119 (reference interval, 62–140) μmol/l) on a biochemistry profile. The horse was then referred for further investigation.

At presentation the horse was quiet but alert and in excess body condition (BCS 4/5 [6]; 681 kg). On auscultation a mild resting tachycardia (48 bpm) was present with no appreciable arrhythmias or murmurs noted. All other vital parameters were within normal limits.

Elevation of haematocrit (47 (reference interval, 31–35) %) was present on a repeat haematology profile. Total serum protein, electrolytes (including total and ionised Ca^2+^, Mg^2+^, Na^+^, Cl^−^ and PO_4_^3−^) and fibrinogen concentrations were within reference range. Cardiac troponin I (CTnI) was markedly elevated (9.82 (reference interval, < 1.3) ng/ml). Blood erythropoietin concentration (5.5, (reference interval, 5.0–10.0) MIU/ml) was within normal limits.

Direct arterial pressure was measured via a catheter in the transverse facial artery. Mean arterial blood pressure was increased (160 mmHg, (reference interval, 80–100) mmHg) with elevation of systolic and diastolic blood pressures (220 and 130 mmHg respectively).

Arterial blood gas analysis was performed prior to, immediately after and 45 min after 15 min of moderate intensity exercise on the lunge. Oxygenation was within normal limits (PaO_2_ 85–95 mmHg) and a mild metabolic acidosis was present immediately after exercise (lactate 3.5, (reference interval <2) mmol/l) that had resolved 45 min later. These findings were considered normal for the fitness of the horse.

**Fig. 1:**
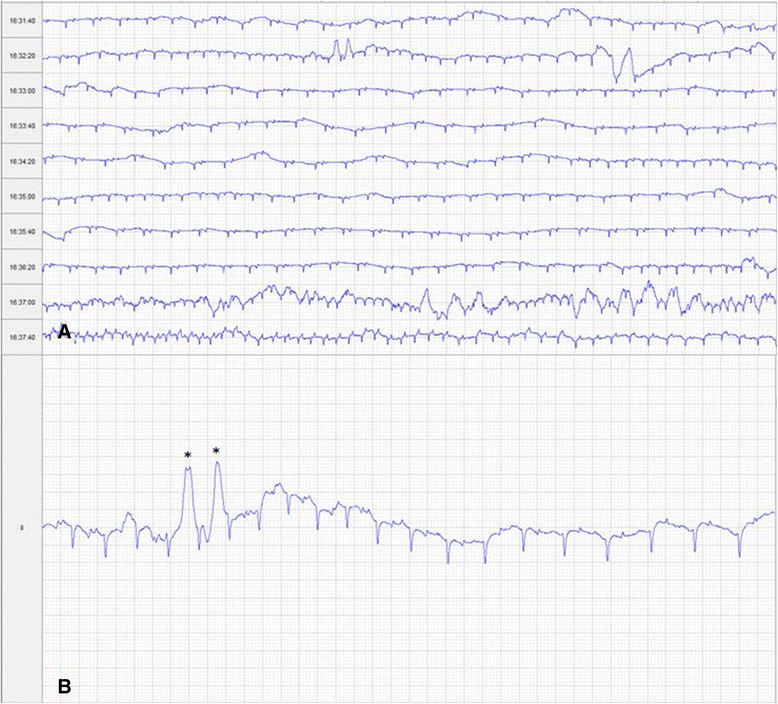
Screen capture ECG traces. **a**. Screen capture ECG trace showing occurrence of multiple VPCs and variable rate within a 6-min period whilst observed resting, undisturbed in stable. The 24-h ECG trace was characterised by multiple runs of resting sinus tachycardia and multiform VPCs (>100). Gain 5 mm/mV, feed 10 mm/s. **b**. Screen capture ECG trace focused on area illustrating two ventricular premature complexes highlighted with an asterix (*). Gain 20 mm/mV, feed 25 mm/s

A telemetric 24-h Holter ECG (Kruuse, Langeskov Denmark) was performed during the period of hospitalisation. Frequent multiform ventricular premature complexes (VPCs) were noted (Fig. 1) with approximately 10 per hour and several short bursts of spontaneous supraventricular tachycardia (maximum HR 180 bpm) were present when the horse was quiet in the stable, each lasting up to 60 s. Abnormal fluctuations in sinus rhythm were appreciable at lower heart rates. Echocardiographic evaluation was impeded by the excess body condition and size of the horse but no significant abnormalities were noted on the images that were obtained.

The findings of a full neurological examination were unremarkable.

An initial diagnosis was made of myocarditis (of unknown aetiology) with syncopal episodes presumed to be due to a temporary reduction in cardiac output and subsequent hypoxia resulting from dysrhythmia. Differentials for the polycythaemia included chronic hypoxaemia and primary polycythaemia (bone marrow neoplasm).

A period of pasture rest was recommended and due to the excess body condition of the horse dietary recommendations (1.5 % of bodyweight of dry hay, soaked for 12 h with a pelleted feed balancer for micronutrient and protein supplementation) were made both for general health and to facilitate further repeat echocardiography.

The horse was re-examined on three further occasions, the first of which was 2 weeks after original discharge. During this 2-week period no further syncopal episodes had been observed and the demeanour of the horse had improved. The resting heart rate had decreased to within normal limits (36–40 bpm). Repeat bloodwork identified a reduction in packed cell volume (37 %) and CTnI (<0.1 (reference interval, <1.3 ng/ml)) was within normal limits. A repeat 24-h telemetric Holter ECG^1^ was unremarkable. This improvement in clinical and clinicopathological parameters supported a resolving acute pathology and a further period of rest was recommended.

A second re-examination was performed 4 months later due to another observed collapse that morning. The horse had been brought in from the field and started eating feed, but then had stopped eating and rested its head against the wall of the stable, with a lowered head carriage and had maintained this position for 20–30 min. After this period the horse had regained normal posture and returned to eating. With the exception of the excess condition of the horse (BCS 4/5; 697 kg) the findings of clinical examination and bloodwork (haematology including acute phase proteins, biochemistry, CTn I) were unremarkable. Repeat echocardiography was again limited by the excess body condition but cardiac chamber dimensions were within normal limits. A telemetric Holter ECG^1^ was placed and a 24-h trace was recorded. The horse was also exercised on the lunge for 10 min at trot and canter. No significant abnormalities were noted.

The final examination was performed just over a year later after a further documented episode of collapse. The horse had been tied up loosely and was eating when it suddenly started to back up with the head raised and subsequently fell down into lateral recumbency. The horse had paddled with the front legs and lip chomped with vacant eyes for approximately 2–3 min, before immediately standing and returning to eating with no suggestion of post-ictal change.

On repeat clinical examination the horse had reduced body condition (BCS 3/5; 613 kg). Vital parameters were within normal limits. Haematology and biochemistry profiles including acute phase proteins and CTnI were all within reference range. Echocardiographic evaluation was again hampered by poor image quality due to the horse’s size, but no abnormalities were noted and measurements taken were within normal limits. A telemetric ECG was placed for 15 h. The majority of the trace was unremarkable with a low resting heart rate (36 bpm), regular rhythm and homogenous morphology of complexes. However, there were multiple short periods of supraventricular tachycardia with no apparent external stimuli as found on previous examination. Ventricular premature complexes were present as previously, but at much reduced frequency (4 VPCs in 15 h).

Due to the intermittent and progressive nature of the syncopal episodes there were significant implications for horse welfare and human safety. Since no treatment options were available the owner elected to have the horse euthanased.

**Fig. 2:**
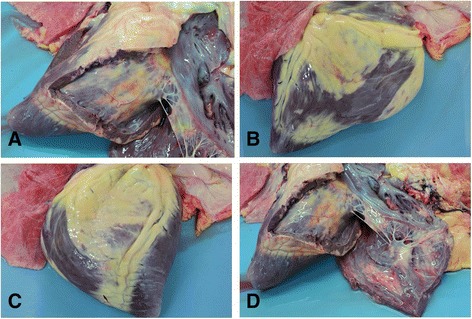
**a**–**d** Four post mortem photographs illustrating gross adipose infiltration of the myocardium of the right ventricle and interventricular septum

On post mortem examination gross and histological abnormalities were restricted to the cardiac muscle. The epicardium of the right ventricular free wall and the endocardium were infiltrated by a yellowish soft tissue extensively expanding deep into the myocardium obliterating more than 60 % of the myofibers. The left ventricle was similarly affected with 20–30 % of the myofibres destroyed by the infiltration. Small foci of this yellowish tissue were also multifocally observed in both the left and right atria (Fig. 2a–d).

**Fig. 3:**
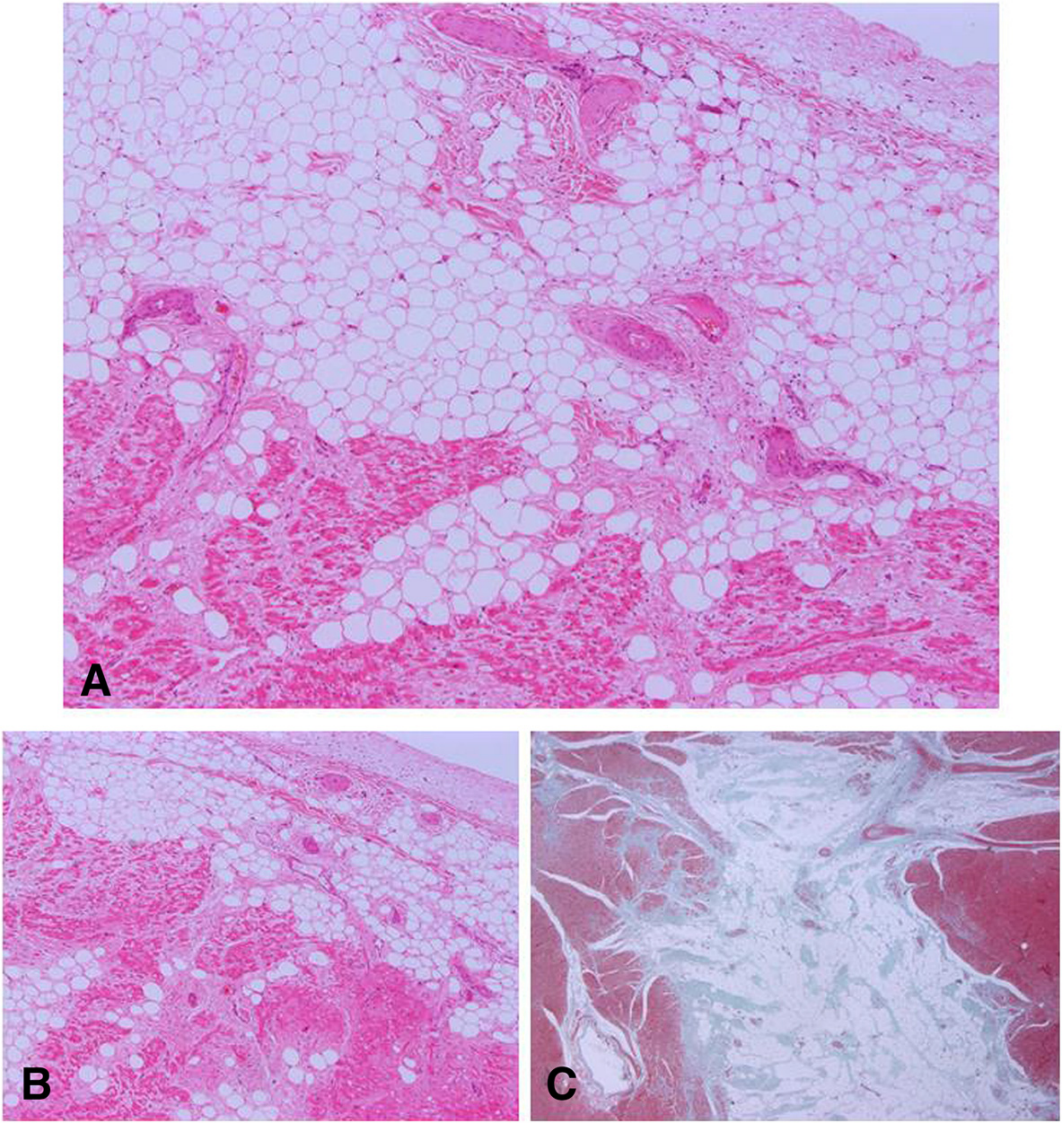
Histological sections of myocardium, endocardium and epicardium. Photomicrographs illustrating a reduction in cardiomyocyte number and replacement with abundant adipose tissue intermixed with variably thickened bundles of fibrous connective tissue. The residual cardiomyocytes are highly vacuolated with loss of myofibrils and nuclear detail. **a** and **b** X40 Objective with Haematoxylin and eosin stain **c**. X12.5 objective with a Masson Trichome stain to highlight fibrosis (green)

Histopathological examination of the myocardium, epicardium and endocardium revealed similar changes in both ventricles. Cardiomyocytes were markedly reduced in number and replaced by abundant adipose tissue intermixed with variably thickened bundles of fibrous connective tissue. Residual cardiomyocytes within or around the adipose tissue were highly vacuolated with loss of the myofibrils and nuclear detail (degeneration). Interspersed were low numbers of lymphocytes and macrophages. Periodic acid-schiff (PAS) stain highlighted the disruption of Purkinje fibres by the adipose tissue. Masson’s trichrome stain highlighted the variably thickened bundles of fibrous connective tissue dissecting the myofibres (Fig. 3a–c). Frozen sections stained with Oil Red confirmed the large vacuolar infiltrate as adipose tissue. There was no evidence of parasitic infiltration grossly or microscopically. The skeletal and smooth muscle were grossly unremarkable in appearance.

A post mortem diagnosis was made of syncope presumed secondary to myocardial degeneration with extensive, severe, chronic, adipose and fibrous infiltration of the right and left ventricle, consistent with arrhythmogenic right ventricular cardiomyopathy.

## Discussion and conclusion

Episodes of collapse are rare in the horse and evidence based literature is limited. The aetiology of the collapse is frequently difficult to establish as it is complicated by the intermittent nature of the clinical signs. In the case series by Lyle et al. [16] only 44 % of horses had a definite diagnosis. The case reported here was classified as syncopal due to the progression of clinical signs to loss of postural tone and full loss of consciousness together with post mortem findings. Differential diagnosis for syncopal collapse can be broadly divided in to cardiac, neurally mediated and miscellaneous causes (volume depletion, endotoxaemia, drugs, dysautonomia) [15].

The abnormalities noted on sequential examinations in this horse pointed to an ante-mortem diagnosis of cardiac pathology. The original presentation with a resting tachycardia, polycythaemia, increased CTnI, frequent VPCs, and intermittent bursts of supraventricular tachycardia was temporally associated with one of the syncopal episodes. Elevated CTnI is a sensitive and specific biomarker of myocardial damage [14]. Boxer dogs with confirmed ARVC had a significantly higher serum CTnI concentration than non-Boxer controls [3] with values correlated to increasing numbers of VPCs per 24 h and grade of ventricular arrhythmia. In horses myocardial disease, structural heart disease and arrhythmias have been reported to cause elevations in CTnI [21]. In this case CTnI was elevated only on first presentation and normalised over 2 weeks, consistent with a discrete insult [22] or more likely in this case, an acute on chronic insult.

Polycythaemia was determined to be absolute (i.e. not secondary to splenic contraction or hypovolaemia) and appropriate due to the repeatability of the abnormality, absence of concurrent markers of hypovolaemia and lack of post mortem renal or neoplastic changes [4]. At the time of sampling the erythropoietin assay was within normal limits (which would support a primary erythrocytosis from bone marrow proliferation) but the half life of erythropoietin is only 5 h [9]. Given the post mortem findings, polycythaemia secondary to chronic hypoxaemia seems more probable. The rapid improvement in red cell count and resting heart rate soon after first evaluation suggest that tissue hypoxaemia was improving at this time.

The relationship between the cardiac pathology noted at post mortem and the syncopal episodes is presumptive but given the cardiac abnormalities on initial examination and absence of other abnormalities on further diagnostics or at post mortem examination, the combined evidence of cardiac syncope is compelling. To form a more definitive ante-mortem diagnosis it would have been beneficial to leave the Holter ECG in place for a longer period in combination with video monitoring when the horse was hospitalised. An implantable cardiac monitor (ICM, ECG recording device) would have allowed longer term monitoring [15], using either a remote, observer operated trigger, or automatic dysrhythmia induced recording.

Fibrofatty infiltration of the cardiac muscle in the horse is not reported as an incidental post mortem finding in reports evaluating a racehorse population [17], working horses [10–12] or a general population [5]. A comparable abattoir study of non athletic or obese horses has not been recorded. Adipose and fibrous tissue infiltration of the myocardium has been documented previously as a cause of cardiac death in two horses [13]. One of these horses presented with ventricular tachycardia that was refractory to medical management and progressed to ventricular fibrillation and death; the second horse was found dead in the field. Both animals had significant adipose and fibrous infiltration of the right ventricle wall and interventricular septum wall with disruption of the Purkinje fibres, similar to this case, and a diagnosis of arrhythmogenic right ventricular cardiomyopathy was made. Unlike this case, the horses reported by Freel et al. [13] both had an enlarged right ventricle, but this may reflect the end progression of the disease, given that both of those cases had suffered fatal cardiogenic collapse. The parallels between these changes and pathologies noted in humans and dogs have been discussed at length previously [13] but it is notable that syncopal episodes are common (52 %) in Boxer dogs with ARVC [1]. Left ventricular pathology which was also noted in this case is observed in a small proportion of affected boxer dogs [20] but greater than 50 % of affected humans [2] with no specific identified risk factors.

This case provides additional evidence of the adverse impact of adipose and fibrous infiltration on cardiac function in the horse and the implications of Purkinje fibre disruption for transmission of electrical activity within the cardiac muscle. Purkinje fibre disruption in the ventricles is associated with the development of ventricular dysrhythmias and sudden death in humans [7].

Clinical manifestations of ARVC in humans relate to development of ventricular tachycardia or ventricular fibrillation and it is a rare but important cause of sudden death in young people, particularly athletes. There is a known genetic susceptibility in 30–50 % of cases [2]. A similar proportion of Boxer dogs have evidence of familial susceptibility with autosomal dominant inheritance suspected [1]. This horse was a 15 year old Clydesdale cross Cob type. The previous report documented a 5 year old Clydesdale and a 15 year old Cob. Further documentation of this rare condition would be required to establish if there is any breed susceptibility.

The excess body condition of the horse may also be pertinent to the development of the cardiac pathology. In humans the relationship between obesity, a pro-inflammatory state, metabolic syndrome and cardiac pathology is well documented and similar infiltrative changes in the myocardium are reported in obese humans [24] as noted in this horesistance seen in Equine Metabolic syndromerse. Increased pericardial adipose tissue deposition is associated with an increased risk of cardiovascular disease, hypothesised to be due to a paracrine effect [8, 18]. It is unknown whether myocardial adipose tissue would have a similar effect in the horse. Endocrine testing was not performed in this horse but obesity is correlated to insulin resistance seen in Equine Metabolic syndrome [26]. Obesity is common in horses in the UK [23, 27] without development of similar clinical signs but in humans cardiovascular disease is multifactorial and the same may be true within the equine population.

In conclusion adipose and fibrous tissue infiltration of the cardiac muscle should be added to the differential diagnosis list as a rare but significant cause of cardiac arrhythmia and syncope when investigating horses for intermittent collapse. This syndrome is potentially comparable to that of arrhythmogenic right ventricular cardiomyopathy noted in boxers and humans and requires further investigation.

## Abbreviations

ARVC: Arrhythmogenic Right Ventricular Cardiomyopathy
CTnI: Cardiac Troponin I
VPC: Ventricular Premature Complex
