# Erratum to: Arrhythmogenic right ventricular cardiomyopathy secondary to adipose infiltration as a cause of episodic collapse in a horse

**DOI:** 10.1186/s13620-017-0099-4

**Published:** 2017-06-07

**Authors:** Alexandra G. Raftery, Nerea Cuesta-Garcia, Hal Thompson, David G. M. Sutton

**Affiliations:** 1 Weipers Centre Equine Hospital, University of Glasgow, Bearsden Road, Glasgow, G611QH UK; 2 Department of Pathology, University of Glasgow, Bearsden Road, Glasgow, G611QH UK

## Erratum

After publication of the original article [1] it was brought to our attention that author Nerea Cuesta-Garcia was incorrectly included as Nuria C. Garcia.

The correct spelling of the name is included in the author list of this erratum.
